# Books and Authors

**Published:** 1911-08

**Authors:** 


### BOOKS AND AUTHORS

**American Practice of Surgery.** A complete System of the Science and Art of Surgery by representative surgeons of the United States and Canada. Edited by **Joseph D. Bryant,** M.D., and **Albert H. Buck,** M.D., New York. Complete in eight volumes. Vol. VIII. Regional Surgery, etc. Imperial octavo, pp. 1146. Profusely illustrated. New York. William Wood & Company. 1909. (Price, cloth, $7.00.)

The present volume completes the largest surgical work in the world's literature, since the days of those prolific and industrious Chinese authors whose writings on medical and allied subjects often filled as many as forty and fifty bulky volumes, and it has become the world's recognised authority on surgical detail.

It is not only difficult but almost impossible adequately to review such a work within the limits at command for there are over one hundred separate contributions from the pens of the most eminent surgeons in this country and Canada, and in simple justice to the excellence of their contributions each should be given that separate consideration demanded by the eminence of the men and the value of their writings. Yet since the work as a whole may be now considered, the salient feature is the pronounced discrimination displayed by the editors in their arrangement of the various subjects and the limitations which they fixed in assigning the work of their collaborators. In no case throughout the entire series of volumes is one impressed with the feeling that here too much space has been given a relatively unimportant subject, and there too little to a surgical condition which should have been more extensively treated. Everywhere is that sense of balance, that indefinable editorial instinct which rounds out and makes for perfect literary architecture. This result were wholly impossible at the hands of a man, however brilliant a surgeon he might be, who had no literary taste; nor could the essential editorial mind, lacking surgical experience have achieved so perfect a goal. There must have been a combination such as controlled the destinies of this great work, an eminent and practical surgeon of authority and literary taste working harmoniously shoulder to shoulder with an editor of widest experience.

This, the last volume contains an article on intrathoracic surgery by the Ransahoffs of Cincinnati, in which they describe the methods of operating in the differential pressure chambers. The surgery of the abdominal organs follows with articles on the spleen by A. E. Garrow, of Montreal; kidneys and ureters by James Bell, of Montreal; pancreas, liver, gall-bladder and biliary passages, by G. D. Stewart, of New York; urinary bladder and prostate, by A. H. Ferguson, of Chicago; ovaries and Fallopian tubes, by R. B. Schenck, of Detroit: uterus and its ligaments, by J. B. Murphy and F. W. Lynch, of Chicago. There are two articles, brief but of exceptional interest, on extrauterine pregnancy and cesarean section and its substitutes contributed by Lewis S. McMurtry, of Louisville. Other chapters concern the law and its relations to the practice of surgery, by Stephen and Sidney Smith, of New York, and Administrative surgical work.

Major Charles Lynch, of the medical department of the United States Army, and editor of *The Military Surgeon,* contributes an interesting article on military surgery which treats of the army organisation, field hospitals, convalescent camps, transportation of the wounded and which is illustrated with a number of photographs of the Japanese military hospitals. This is followed by a similar article by Surgeon General Stokes of the navy, dealing with the difficulties of caring for the wounded at sea during an engagement.

In the appendix, the relation of blood pressure to surgery, is handled by J. E. Sweet, of Philadelphia in an excellent manner.

The publishers are to be congratulated on their portion of the work. The illustrations are not surpassed in any printed book and the typographic excellence is unusual and wholly beyond any honest criticism.

---

**Plaster of Paris and How to Use it.** By **Martin W. Ware,** M.D., Instructor of Surgery in the New York Post-Graduate Medical School. Second edition. New York: Surgery Publishing Co. (Cloth, $1.25.)

There are few medical books published of such irresistible appeal to the general practitioner as this exceptional and valuable little work. The first edition was soon exhausted and the necessity for a second issue gave Dr. Ware opportunity to revise and practically rewrite a large portion of it. In its enlarged form its usefulness has been greatly enhanced. New drawings and marginal side notes in red have been added and there are now ninety illustrations which are of definite help in grasping the fundamental principles of the book. Under the general heading of Plaster of Paris bandages there are sections devoted to history, materials used in manufacture of bandages, storage, the bandages of commerce, the immediate preparation of bandages and the Calot plaster bandages. There are also chapters dealing with the application of the plaster bandage to individual fractures, fractures of the upper extremity, the lower extremity, molded plaster, splints and plaster in orthopedic surgery. These are presented in such a comprehensive manner and in such understandable language that the book, small though it be, is one of the really invaluable contributions to medical literature. The publishers have ably seconded Ware's efforts to give the profession a book of distinct merit.

**Golden Rules of Pediatrics.** Practical Rules for Diagnosis and Prognosis. Essentials for Feeding. Principles of Scientific Treatment. By **John Zahorsky,** A.B., M.D., Clinical Professor of Pediatrics, Medical Department of Washington University. With introduction by **E. W. Saunders,** M.D. Second edition, revised and enlarged. St. Louis, Mo.: C. V. Mosby Co. (Price, $2.50 net.)

The Golden Rule Series has established itself as one of the most valuable of its kind. This is evinced in that a second edition is being sent out of this particular book and, already, several of the set are either on the market or in process of republication.

Professor Zahorsky has devoted considerable time to the revision now presented and in making it he has incorporated in the text considerable matter which is absolutely new to it. The volume as it now stands is considerably increased in value and will interest not only new readers, but those who may have the good fortune to possess the first edition as well.

**The Practical Medicine Series.** Ten Volumes. Under the general editorial charge of **Gustavus P. Head,** M.D. Vols. I, II and III. General Medicine. Edited by **Frank Billings,** M.D. and **J. H. Salisbury,** M.D. Vol. II. General Surgery. Edited by **John B. Murphy,** A.M., M.D., LL.D., Professor of Surgery in Northwestern University. Vol. III. **Eye, Ear, Nose and Throat.** Edited by **Casey A. Wood,** M.D., **Albert H. Andrews,** M.D., **G. P. Head,** M.D. Series 1911. Chicago: The Year Book Publishers. (Prices, $1.50, $2.00; entire series, $10.00.)

These three volumes show continued excellence of the series and there is nothing lacking in them which would tend to make for their completeness. The volume on eye, ear, nose and throat was prepared by C. A. Wood, Albert H. Anderson and Gustavus P. Head. It is excellently illustrated and most complete in its exposition of the specialties dealt with. General medicine, under the supervision of Frank Billings and J. H. Salisbury is without flaw. There is special stress laid upon diagnosis. Dr. John B. Murphy had prepared the volume on general surgery and as a result it is one of the most complete of the series. With Murphy's well-known careful attention to detail the volume rests well within the lines of perfection.

---

**1,000 Surgical Suggestions.** By **Walter M. Brickner,** B.S., M.D., Adjunct Surgeon Mount Sinai Hospital, Editor in Chief **"American Journal of Surgery."** Fourth edition. 12 mo. pp. 225. New York: Surgery Publishing Co. 397 pages. (Cloth, $1.00.)

The first edition of this handy little volume was issued in 1906 and since then four issues have been necessary. It has been thoroughly revised and there has been a considerable enlargement of the material contained in its pages. Over 300 suggestions have been added. It is excellently put together and will be of value.

---

**Health Hints.** By **E. R. Pritchard,** Secretary of the Chicago Department of Health. Small 12 mo, pp. 153. Chicago: The Reilly & Britton Co. (Cloth, 50 cents.)

Although there are only 154 pages in this little book it is of great use written as it is in simple language and designed to be of especial value to the people at large. It discusses air and breathing, cleanliness, contagion, consumption, eating and digestion, food for infants, infections, light, rest and sleeping, out of doors life, sanitation, water and the care of school children.

---

**Tuberculosis, as a Disease of the Masses, and How to Combat It.** By **S. A. Knopf,** M.D. Awarded the International Prize by the Committee of the International Congress to Combat Tuberculosis as a Disease of the Masses, which convened at Berlin, May 24-27, 1909. Seventh edition. New York: The Survey; also for sale by **F. P. Flori,** New York. 1911. (Paper, 25 cents.) ,

The above data are the best credentials of this work, which is recognised as authoritative.

